# Genomic Characterization of an O101:H9-ST167 NDM-5-Producing Escherichia coli Strain from a Kitten in Italy

**DOI:** 10.1128/spectrum.00832-22

**Published:** 2022-06-06

**Authors:** Gherard Batisti Biffignandi, Aurora Piazza, Federica Marchesini, Paola Prati, Alessandra Mercato, Aseel Abu Alshaar, Giuseppina Andreoli, Davide Sassera, Roberta Migliavacca

**Affiliations:** a Department of Clinical-Surgical, Diagnostic and Pediatric Sciences, University of Pavia, Pavia, Italy; b Department of Biology and Biotechnology “L. Spallanzani”, University of Pavia, Pavia, Italy; c Istituto Zooprofilattico Sperimentale della Lombardia e dell'Emilia Romagna “B. Ubertini”, Pavia, Italy; Institut National de Santé Publique du Québec

**Keywords:** NDM-5, *Escherichia coli*, ST167, plasmid, WGS, hybrid genome, phylogeny, One-Health, companion animals, multidrug resistance, plasmid analysis

## LETTER

The high-risk clone ST167 associated with *bla*NDM-5 resistance determinant is currently recognized to be a source of public health concern worldwide (1–4), since it has been identified even beyond hospital borders, in companion animals, wastewater, rivers, and wildlife (5–8).

In this work, we characterized an NDM-5-producing Escherichia coli ST167 collected in Italy from a liver sample of a 4-month-old cat who died from parvovirus hemorrhagic enteritis. The E. coli strain 167624 was tested for antibiotic susceptibility and sequenced using both Illumina and Nanopore technologies.

Bacterial identification and antibiotic susceptibility tests were performed with the semiautomated system MicroScan autoSCAN4 (Beckman Coulter); results were interpreted according to EUCAST guidelines (v10.0-2020, http://www.eucast.org). The E. coli 167624 strain showed a multidrug-resistant (MDR) profile, being resistant to all the antibiotics tested, with the exception of colistin, amikacin, and fosfomycin (Table 1).

**TABLE 1:** Antimicrobial susceptibility profile of the ECO167624 strain

Antibiotic	MIC^*a*^ (μg/mL)	Interpretation
AMK	≤8	S
AMP	>8	R
AMC	>8|4	R
AZT	>4	R
FEP	>8	R
CTX	>16	R
CAZ	>8	R
CIP	>1	R
LEV	>1	R
GNT	>4	R
COL	≤2	S
FOS	≤32	S
ERT	>1	R
MER	>8	R
PTZ	>16	R
SXT	>4|76	R
PIP	>16	R
TBR	>4	R

a AMK, amikacin; AMP, ampicillin; AMC, amoxicillin/clavulanate; AZT, aztreonam; FEP, cefepime; CTX, cefotaxime; CAZ, ceftazidime; CIP, ciprofloxacin; LEV, levofloxacin; GNT, gentamicin; COL, colistin; FOS, fosfomycin; ERT, ertapenem; MER, meropenem; PTZ, piperacillin-tazobactam; SXT, trimethoprim-sulfamethoxazole; PIP, piperacillin; TBR, tobramycin; S, susceptible; R, resistant. Susceptibility results were interpreted according to the European Committee on Antimicrobial Susceptibility Testing (EUCAST, 2020) criteria.

**FIG 1:**
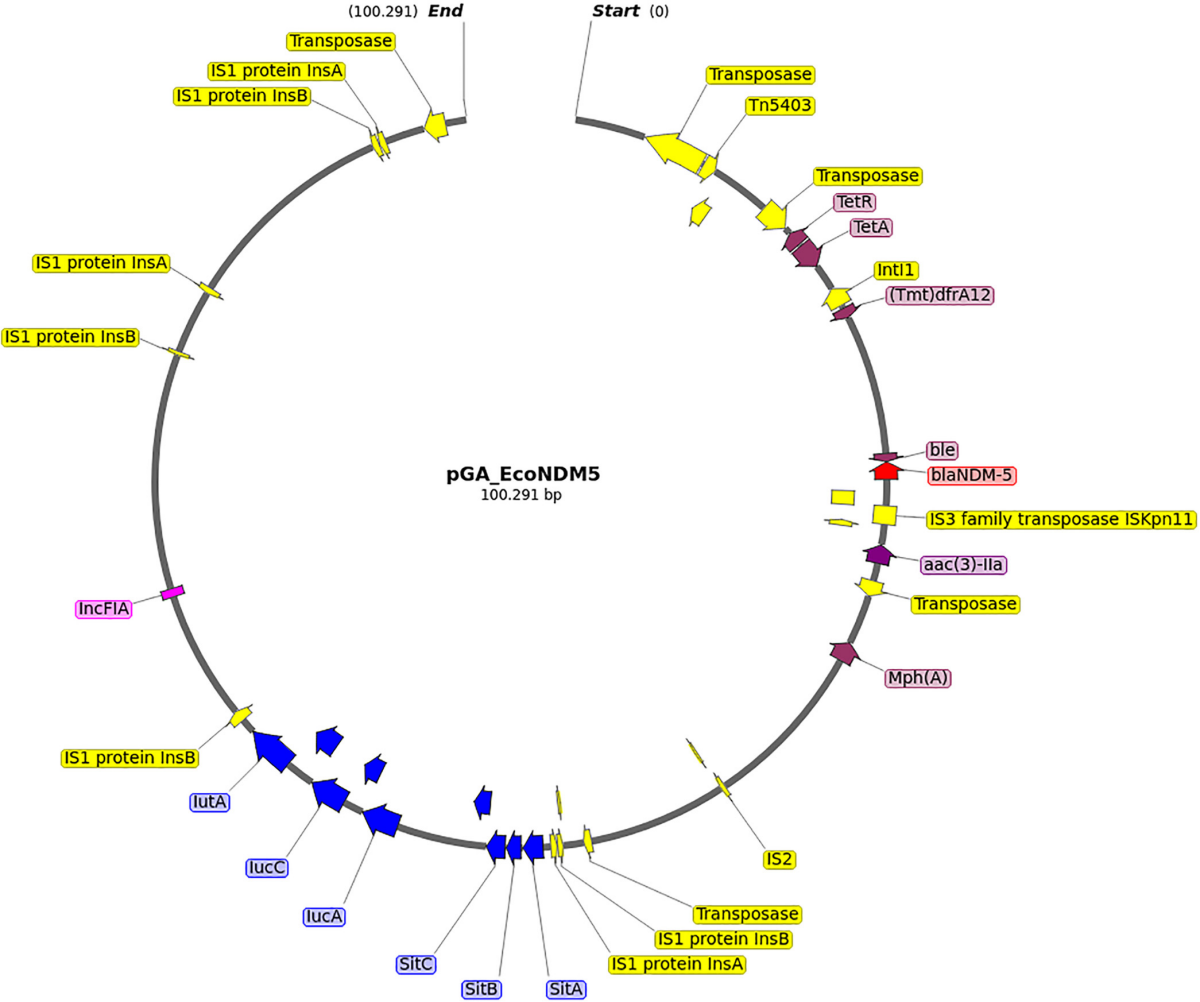
Graphical representation of the pGA_EcoNDM5 plasmid sequence. Colored arrows represent genes or coding regions: red, *bla*NDM-5 gene; purple, antimicrobial resistance genes; yellow, insertion sequences (IS) and transposons; blue, aerobactin operon and virulence genes; fuchsia, incompatibility group.

Genomic DNA was sequenced via both Oxford Nanopore MinION, with library FLO-MIN106 (rapid barcoding kit SQK-RBK004), and Illumina MiSeq platform (Nextera XT library preparation kit, with a 2 × 250 paired-end run), after extraction with DNeasy blood and tissue kit (Qiagen). A complete hybrid genome was obtained (genome size of 5,141,416 bp, chromosome sequence of 4,849,672 bp) using Unicycler v0.4.8-beta (9). A main plasmid, pGA_EcoNDM5 (size of 100,291 bp), harboring the *bla*NDM-5 gene was detected and annotated (Fig. 1, and see supplemental material).

*In silico* multilocus sequence type (MLST) analysis showed that the strain ECO16724 belonged to the high-risk clone ST167 (MLST Achtman scheme), phylogroup A, and serotype O101:H9.

Investigation of the resistance genes content highlighted the copresence of multiple β-lactamase determinants, including the plasmid-borne *bla*NDM-5 and *bla*ble, as well as *bla*AmpH and *bla*AmpC1 on the chromosome. In addition, virulence factors associated with flagellar motility (*Fli/Flg* family), fimbriae (*fimF*, *fimG*), and siderophore (*ybtT*, *iucA*) were detected on the chromosome and on the pGA_NDM5 plasmid. Resistance determinants included *bla*NDM-5, *bla*ble, *bla*AmpH, *bla*AmpC1, *gyrA* (S83L, D87N), *parC* (S80 I), *parE* (S458A), *mph(A)*, *tet(A)*, *tet(R)*, *aac(3)-Ila*, *aadA2*, *sul1*, and *dfrA12*. Virulence determinants included *fliN*, *fliM*, *fliL*, *fliJ*, *fliA*, *flgH*, *flgG*, *flgD*, *flgC*, *flgB*, *fimF*, *fimG*, *ybtT*, *iucA*, *cea*, *capU*, *fyuA*, *gad*, *hra*, *irp2*, and aerobactin operon. The pGA_EcoNDM5 belonged to the IncFIA with an identity score of 99.48%.

**FIG 2:**
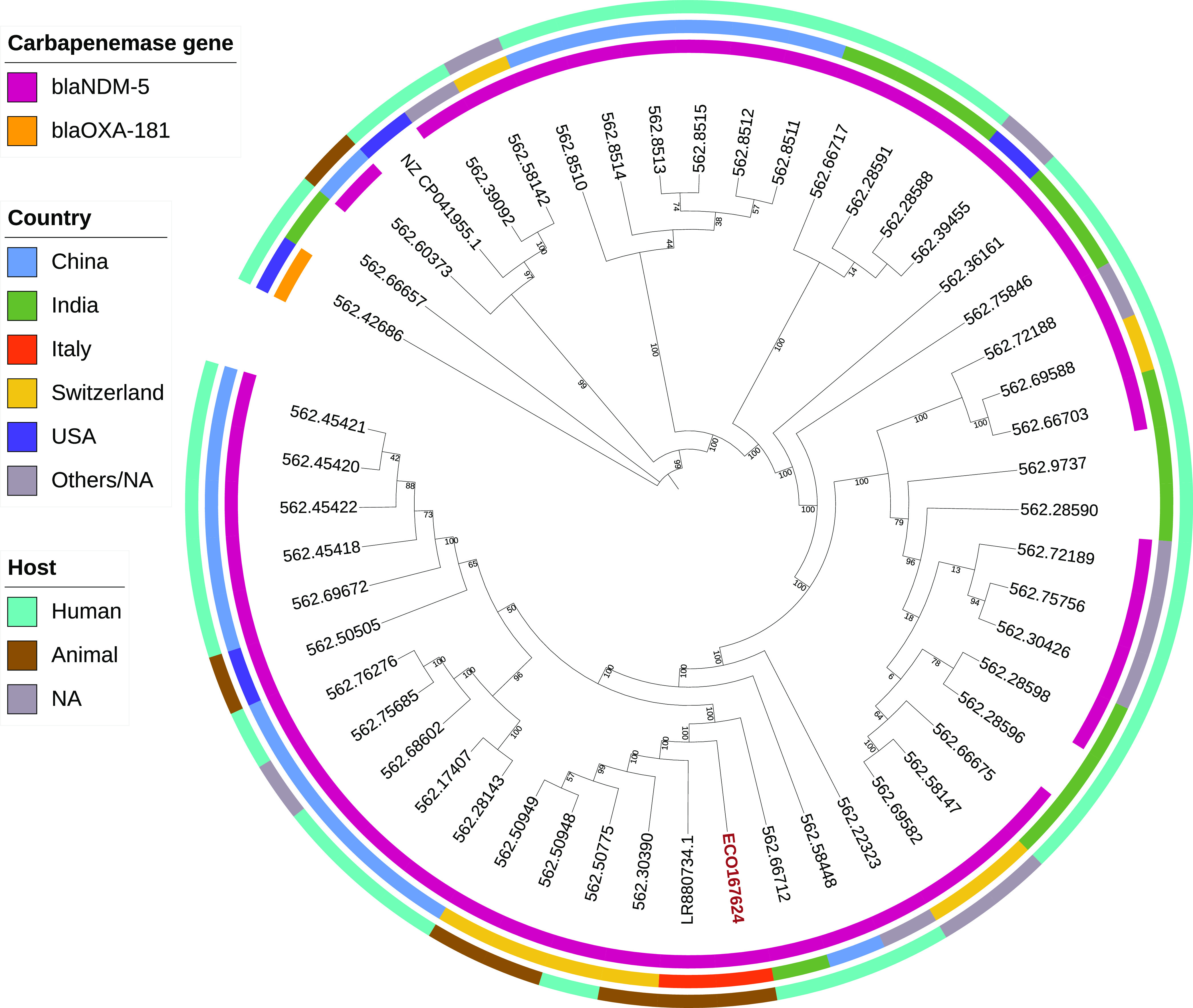
CoreSNP-based phylogeny of the 50 E. coli strains closest to ECO167624 retrieved from PATRIC database.

To place the ECO16724 isolate within the proper taxonomic context, a coreSNP phylogeny was inferred (see supplemental material). The phylogenetic analysis (Fig. 2) showed ECO167624 to be part of a clade including *bla*NDM-5-positive strains: four from human and dog sources in Switzerland (2017 to 2018) and one, LR880734.1, from a dog in Italy (2019).

The comparison of the *bla*NDM-5 genetic environment among the plasmids of the strains within this clade highlighted a high similarity, showing the same NDM-carrying integron (Fig. S1).

Transmission between animals and humans of ST167 NDM-5-producing E. coli has been already demonstrated in a familiar context (7). Although we were not able to trace the origin of the here-presented ECO167624 strain, a human-animal transmission event could be hypothesized. In Italy, the *bla*NDM-5 gene is to date associated mainly with human clinical cases (1, 2), but our results raise the hypothesis that community could represent a hidden reservoir of NDM-5-producing ST167 high-risk clone.

The ability to trace rapidly the source of infection is of particular relevance in a globalized world, where the boundaries among the different settings (humans, environment, animals) are continuously crossed by bacteria. Hence, the standardization of tools and user-friendly platforms for the genomic surveillance, such as Pathogenwatch and BacWGSTdb 2.0 (10, 11), is acquiring an increasingly pivotal role.

The increased reports of MDR clones in the hospital, community, and environment surely sound like an alarm bell, suggesting the appropriateness of the “One-Health” approach.

### Data availability.

The nucleotide sequence of the strain ECO167624 was submitted to NCBI with the following accession codes: BioProject ID PRJNA816063 and BioSample SAMN26656496.

## References

[1] Corbellini S, Scaltriti E, Piccinelli G, Gurrieri F, Mascherpa M, Boroni G, Amolini C, Caruso A, De Francesco MA. 2022. Genomic characterisation of *Escherichia coli* isolates co-producing NDM-5 and OXA-1 from hospitalised patients with invasive infections. J Glob Antimicrob Resist 28:136–139. doi:10.1016/j.jgar.2021.12.018. 34965471

[2] Giufrè M, Errico G, Accogli M, Monaco M, Villa L, Distasi MA, Del Gaudio T, Pantosti A, Carattoli A, Cerquetti M. 2018. Emergence of NDM-5-producing *Escherichia coli* sequence type 167 clone in Italy. Int J Antimicrob Agents 52:76–81. doi:10.1016/j.ijantimicag.2018.02.020. 29501819

[3] Weingarten RA, Johnson RC, Conlan S, Ramsburg AM, Dekker JP, Lau AF, Khil P, Odom RT, Deming C, Park M, Thomas PJ, Henderson DK, Palmore TN, Segre JA, Frank KM, NISC Comparative Sequencing Program. 2018. Genomic analysis of hospital plumbing reveals diverse reservoir of bacterial plasmids conferring carbapenem resistance. mBio 9:e02011-17. doi:10.1128/mBio.02011-17. 29437920 PMC5801463

[4] Zou H, Jia X, Liu H, Li S, Wu X, Huang S. 2020. Emergence of NDM-5-producing *Escherichia coli* in a teaching hospital in Chongqing, China: IncF-Type plasmids may contribute to the prevalence of *bla*NDM-5. Front Microbiol 11:334. doi:10.3389/fmicb.2020.00334. 32210935 PMC7069339

[5] Bleichenbacher S, Stevens MJA, Zurfluh K, Perreten V, Endimiani A, Stephan R, Nüesch-Inderbinen M. 2020. Environmental dissemination of carbapenemase-producing *Enterobacteriaceae* in rivers in Switzerland. Environ Pollut 265:115081. doi:10.1016/j.envpol.2020.115081. 32806462

[6] Alba P, Taddei R, Cordaro G, Fontana MC, Toschi E, Gaibani P, Marani I, Giacomi A, Diaconu EL, Iurescia M, Carfora V, Franco A. 2021. Carbapenemase IncF-borne *bla*NDM-5 gene in the *E. coli* ST167 high-risk clone from canine clinical infection, Italy. Vet Microbiol 256:109045. doi:10.1016/j.vetmic.2021.109045. 33887564

[7] Grönthal T, Österblad M, Eklund M, Jalava J, Nykäsenoja S, Pekkanen K, Rantala M. 2018. Sharing more than friendship - transmission of NDM-5 ST167 and CTX-M-9 ST69 *Escherichia coli* between dogs and humans in a family, Finland, 2015. Euro Surveill 23:1700497. doi:10.2807/1560-7917.ES.2018.23.27.1700497. PMC6152158 29991384

[8] Peterhans S, Stevens M, Nüesch-Inderbinen M, Schmitt S, Stephan R, Zurfluh K. 2018. First report of a *bla*NDM-5-harbouring *Escherichia coli* ST167 isolated from a wound infection in a dog in Switzerland. J Glob Antimicrob Resist 15:226–227. doi:10.1016/j.jgar.2018.10.013. 30339894

[9] Wick RR, Judd LM, Gorrie CL, Holt KE. 2017. Unicycler: resolving bacterial genome assemblies from short and long sequencing reads. PLoS Comput Biol 13:e1005595. doi:10.1371/journal.pcbi.1005595. 28594827 PMC5481147

[10] Argimón S, David S, Underwood A, Abrudan M, Wheeler NE, Kekre M, Abudahab K, Yeats CA, Goater R, Taylor B, Harste H, Muddyman D, Feil EJ, Brisse S, Holt K, Donado-Godoy P, Ravikumar KL, Okeke IN, Carlos C, Aanensen DM, NIHR Global Health Research Unit on Genomic Surveillance of Antimicrobial Resistance. 2021. Rapid genomic characterization and global surveillance of *Klebsiella* using Pathogenwatch. Clin Infect Dis 73:S325–S335. doi:10.1093/cid/ciab784. 34850838 PMC8634497

[11] Feng Y, Zou S, Chen H, Yu Y, Ruan Z. 2021. BacWGSTdb 2.0: a one-stop repository for bacterial whole-genome sequence typing and source tracking. Nucleic Acids Res 49:D644–D650. doi:10.1093/nar/gkaa821. 33010178 PMC7778894

