# Supplementary material for: Genomic Characterization of an O101:H9-ST167 NDM-5-Producing Escherichia coli Strain from a Kitten in Italy
Source: Microbiol Spectr. 2022 Jun 6;10(3):e00832-22. doi: 10.1128/spectrum.00832-22 (PMC9241605; doi:10.1128/spectrum.00832-22)
Supplement: Supplemental file 1 — Supplemental material. Download spectrum.00832-22-s0001.pdf, PDF file, 0.5 MB [file spectrum.00832-22-s0001.pdf]

Supplementary

Genomic typing: resistome, virulome, serotyping and phylogroup

The MultiLocus Sequence Type (MLST) of the ECO16764 strain was assessed using the PubMLST web-server (1) and the Achtman scheme. The resistance and virulence genes content was determined with Abricate v0.9.8 (https://github.com/tseemann/abricate), considering the ResFinder (2) and VirulenceFinder databases (3,4). The presence of a resistance gene was determined considering only the exact matches. Virulence genes with at least 90% coverage and 90% identity were selected. Plasmid sequences were screened using both Plasmid Finder (5) and NCBI database annotations using BLASTn, looking at the hits of sequences external to the chromosome. In addition to the main plasmid PGA_EcoNDM-5, five other external sequences were generated by the hybrid assembly, four of them circular and one linear, with the following lengths: 90.296bp, 76.841bp, 11.208bp, 8.335bp and 4.773bp.
The main plasmid PGA_EcoNDM-5 has shown its best hit against the plasmid CP043230.1 (identity of 99.74%, coverage 100%). The other best hits were respectively: LR880736.1 (identity of 100%, coverage 100%), CP057691.1 (identity of 98.15%, coverage 76%), MH985167.1 (identity 99.97%, coverage 61%) with InCFIB, CP016513.1 (identity 99.98%, coverage 79%) and CP082631.1 (identity 99.43%, coverage 100%).

Serotyping and Phylogroup were determined using SerotypeFinder (6) and ClermontTyping (7) softwares respectively. Genome annotation was performed using Prokka-v1.14.6 (8).

Phylogenetic analysis

All the available *Escherichia coli* genomes were downloaded from the PATRIC database, updated to July 2021 (9). Genomes with the genome size > 4Mb, number of contigs < 300 and N50 > 50.000bp were selected. The genetic distance based on *k*-mers between the ECO16724 and all the selected strain from the PATRIC was calculated using Mash algorithm (10). The first 50 closest hits were considered. All the 50 selected assemblies were aligned to a complete reference genome (NZ_CP041955.1) using progressiveMauve (11). Then, the coreSNPs were called using the software Purple, described by Gona and colleagues (12). Core SNPs are considered as single-nucleotide variable sites flanked by at least five conserved bases present in all the selected genomes of this analysis.

The coreSNPs alignment was used to infer the phylogenetic analysis through the RAxML software v8.2.8 (13). The evolutionary model ASC_GTRGAMMA was chosen, considering the Lewis correction (14) and 100 bootstrap replicates.

Plasmid map and comparison

The annotations of the plasmid content were generated using Prokka v1.14.6 (8). Plasmid map was designed using SnapGene viewer software (snapgene.com).

The plasmids sequences of the strains closest to ECO167164 including the latter were aligned using progressiveMauve (11) and visualised using R software v3.6.3 (15) through the package genoPlotR (16).

Reference

1. Jolley, K. A., Bray, J. E., & Maiden, M. C. J. 2018. Open-access bacterial population genomics: BIGSdb software, the PubMLST.org website and their applications. *Wellcome Open Research*, 3, 124.
2. Bortolaia, V., Kaas, R. S., Ruppe, E., Roberts, M. C., Schwarz, S., Cattoir, V., Philippon, A., Allesoe, R. L., Rebelo, A. R., Florensa, A. F., Fagelhauer, L., Chakraborty, T., Neumann, B., Werner, G., Bender, J. K., Stingl, K., Nguyen, M., Coppens, J., Xavier, B. B., Aarestrup, F. M. 2020. ResFinder 4.0 for predictions of phenotypes from genotypes. *Journal of Antimicrobial Chemotherapy*, 75(12), 3491–3500.
3. Joensen, K. G., Scheutz, F., Lund, O., Hasman, H., Kaas, R. S., Nielsen, E. M., & Aarestrup, F. M. 2014. Real-time whole-genome sequencing for routine typing, surveillance, and outbreak detection of verotoxigenic *Escherichia coli*. *Journal of Clinical Microbiology*, 52(5), 1501–1510.
4. Malberg Tetzschner, A. M., Johnson, J. R., Johnston, B. D., Lund, O., & Scheutz, F. 2020. In Silico Genotyping of Escherichia coli Isolates for Extraintestinal Virulence Genes by Use of Whole-Genome Sequencing Data. *Journal of Clinical Microbiology*, 58(10), e01269-20.
5. Carattoli, A., Zankari, E., García-Fernández, A., Voldby Larsen, M., Lund, O., Villa, L., Møller Aarestrup, F., & Hasman, H. 2014. *In silico* detection and typing of plasmids using PlasmidFinder and plasmid multilocus sequence typing. *Antimicrobial Agents and Chemotherapy*, 58(7), 3895–3903.
6. Joensen, K. G., Tetzschner, A. M., Iguchi, A., Aarestrup, F. M., & Scheutz, F. 2015. Rapid and Easy *In Silico* Serotyping of *Escherichia coli* Isolates by Use of Whole-Genome Sequencing Data. *Journal of Clinical Microbiology*, 53(8), 2410–2426.
7. Beghain, J., Bridier-Nahmias, A., Le Nagard, H., Denamur, E., & Clermont, O. 2018. ClermonTyping: an easy-to-use and accurate *in silico* method for *Escherichia* genus strain phylotyping. *Microbial Genomics*, 4(7), e000192.
8. Seemann T. (2014). Prokka: rapid prokaryotic genome annotation. *Bioinformatics* (Oxford, England), 30(14), 2068–2069.
9. Davis, J. J., Wattam, A. R., Aziz, R. K., Brettin, T., Butler, R., Butler, R. M., Chlenski, P., Conrad, N., Dickerman, A., Dietrich, E. M., Gabbard, J. L., Gerdes, S., Guard, A., Kenyon, R. W., Machi, D., Mao, C., Murphy-Olson, D., Nguyen, M., Nordberg, E. K., Olsen, G. J. Stevens, R. 2020. The PATRIC Bioinformatics Resource Center: expanding data and analysis capabilities. *Nucleic Acids Research*, 48(D1), D606–D612.
10. Ondov, B. D., Treangen, T. J., Melsted, P., Mallonee, A. B., Bergman, N. H., Koren, S., & Phillippy, A. M. 2016. Mash: fast genome and metagenome distance estimation using MinHash. *Genome Biology*, 17(1), 132.

11. Darling, A. E., Mau, B., & Perna, N. T. 2010. progressiveMauve: multiple genome alignment with gene gain, loss and rearrangement. *PloS one*, 5(6), e11147.
12. Gona, F., Comandatore, F., Battaglia, S., Piazza, A., Trovato, A., Lorenzin, G., Cichero, P., Biancardi, A., Nizzero, P., Moro, M., & Cirillo, D. M. 2020. Comparison of core-genome MLST, coreSNP and PFGE methods for *Klebsiella pneumoniae* cluster analysis. *Microbial Genomics*, 6(4), e000347.
13. Stamatakis, A. 2014. RAxML version 8: a tool for phylogenetic analysis and post-analysis of large phylogenies. *Bioinformatics*, 30(9), 1312–1313.
14. Lewis, P. O. 2001. A likelihood approach to estimating phylogeny from discrete morphological character data. Systematic Biology, 50(6), 913–925.
15. R Core Team 2021. R: A language and environment for statistical computing. R Foundation for Statistical Computing, Vienna, Austria.URL https://www.R-project.org/.
16. Guy, L., Roat Kultima, J., & Andersson, S. G. E. 2010. genoPlotR: comparative gene and genome visualization in R. In *Bioinformatics* (Vol. 26, Issue 18, pp. 2334–2335).

Supplementary Fig. S1. Graphical Mauve representation of the regions of homology in the *bla*NDM-5-harbouring plasmids in the ECO16724 isolate and its five phylogenetically closest relatives.

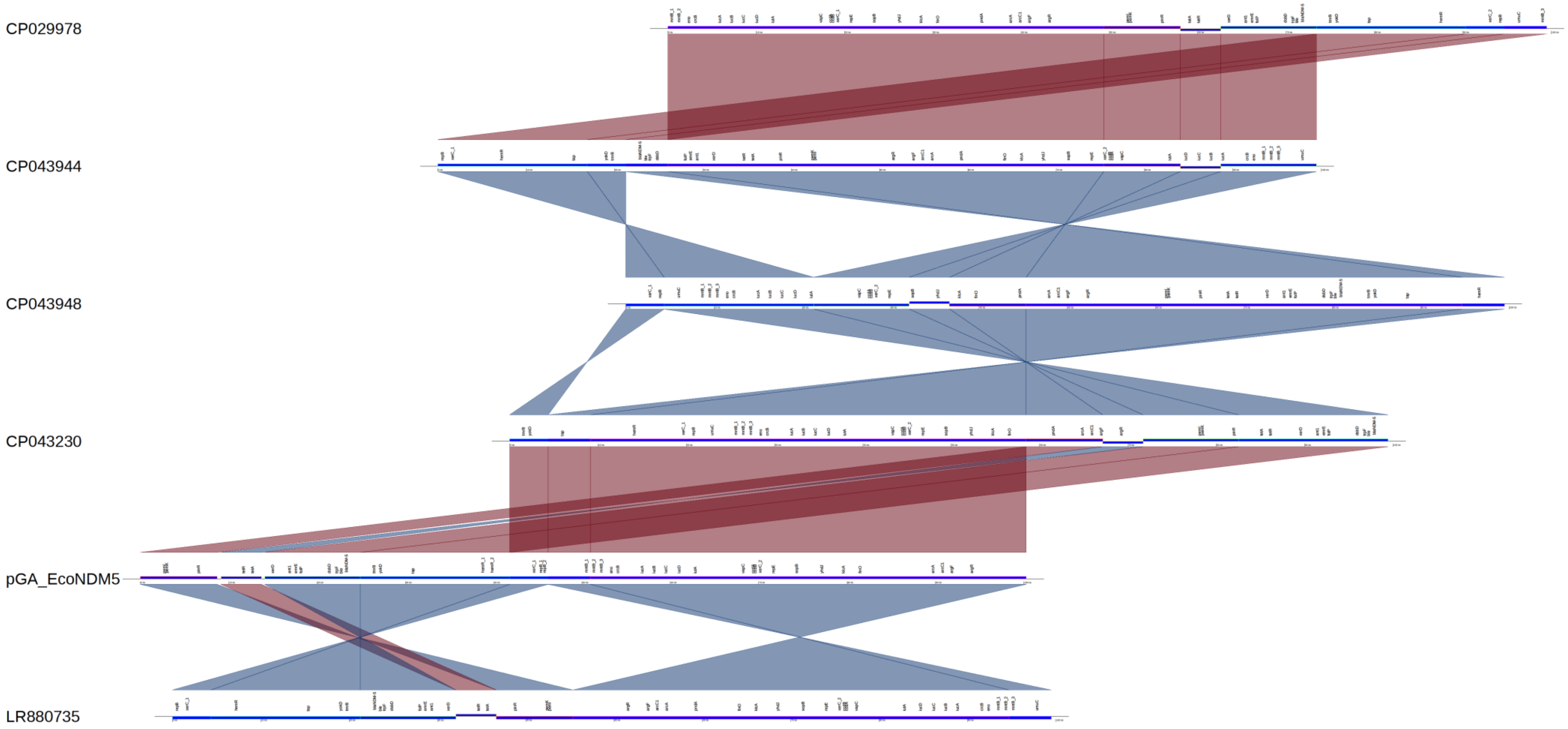

Supplementary Fig. S1
